# Serum creatinine/cystatin C ratio as a screening tool for sarcopenia and prognostic indicator for patients with esophageal cancer

**DOI:** 10.1186/s12877-022-02925-8

**Published:** 2022-03-15

**Authors:** Chao Zheng, Ellen Wang, Jiang-Shan Li, Kai Xie, Chao Luo, Qi-Yue Ge, Li-Wen Hu, Yi Shen

**Affiliations:** 1 Department of Cardiothoracic Surgery, Jinling Hospital, School of Medicine, Southeast University, Nanjing, China; 2 McMaster University, Hamilton, Canada; 3 University of Science and Technology Beijing, Beijing, China; 4 Department of Cardiothoracic Surgery, Jinling Hospital, Jinling School of Clinical Medicine, Nanjing Medical University, Nanjing, China; 5 Department of Cardiothoracic Surgery, Jinling Hospital, Southern Medical University, Guangzhou, China; 6 Department of Cardiothoracic Surgery, Jinling Hospital, School of Medicine, Nanjing University, Nanjing, China

**Keywords:** Sarcopenia index, NHANES, Esophageal cancer, Sarcopenia, Prognosis

## Abstract

**Background & aims:**

Sarcopenia is associated with poor clinical outcomes of patients who underwent esophagectomy. The current diagnostic criteria for sarcopenia are complex and laborious. We aimed to employ the simple and economic indicator sarcopenia index (SI = creatinine/cystatin C ×100) to screen for sarcopenia and to evaluate its prognostic value in patients with esophageal cancer (EC).

**Methods:**

Older participants in the National health and nutrition examination survey (NHANES) database (1999–2002) were divided into three groups according to tertiles of the SI value to explore the feasibility of SI in the diagnosis of sarcopenia. Restricted cubic spline (RCS) was utilized to show the non-linear relationship between all-cause mortality and SI. Patients with EC admitted to Jinling Hospital were enrolled to validate the efficacy and prognostic value of SI. Cut-off values of SI were determined using receiver operating characteristic curves. Multivariable logistic analyses and Cox analyses were used to identify the independent factors of postoperative complications and long-term survival, respectively.

**Results:**

A total of 989 participants were identified from the NHANES database. SI showed the diagnostic value of sarcopenia (tertile 1 vs. tertile 3: odds ratio [OR]=3.67, 95% confidence interval [CI]: 1.52–8.87, *p*=0.004; tertile 2 vs. tertile 3: OR=1.79, 95% CI: 0.75–4.28, *p*=0.191) adjusted for race, gender, and body mass index (BMI). Individuals with SI ≤ 68 had a poorer overall survival (OS) (hazard ratio [HR]=2.14, 95% CI: 1.71–2.68, *p*<0.001), and the RCS plot showed that the all-cause mortality risk gradually decreased with the increase in SI. Then, 203 patients with EC were enrolled, of which 76 patients were diagnosed with sarcopenia. There was a linear correlation between SI and skeletal muscle index and prealbumin, indicating that SI was reliable for diagnosing sarcopenia. Patients in the high sarcopenia risk group (Male: SI < 62; Female: SI < 55) showed a higher incidence of complications (OR=3.50, 95% CI: 1.85–6.61, *p*<0.001) and poorer long-term survival (HR=2.62, 95% CI: 1.02–6.77, *p*=0.046).

**Conclusion:**

SI could be used to identify sarcopenia in patients with EC, and it is a useful prognostic factor of postoperative complications and long-term survival.

**Supplementary Information:**

The online version contains supplementary material available at 10.1186/s12877-022-02925-8.

## Introduction

Esophageal cancer (EC) is an important health burden worldwide, with high morbidity and mortality [1]. Due to factors, such as advanced age, nutritional limitations, and tumor cachexia, the prevalence of sarcopenia is high in EC patients, with a rate of 14.4%–80% [2]. Sarcopenia is defined as an age-related, progressive, and widespread disorder with reduced skeletal muscle mass or muscle strength. In recent years, sarcopenia has received widespread attention as it is associated with an increased likelihood of adverse outcomes, such as falls, fractures, physical disability, and death among the older adults. It has been reported that sarcopenia is not only a predictor of postoperative complications in patients with EC but is also detrimental to long-term survival [3]. Therefore, early identification of sarcopenia in patients with EC is important.

Based on the existing international consensus [4–6], diagnosis of sarcopenia requires an assessment of muscle mass, muscle strength, and physical performance. The most reliable tools for measuring muscle mass include dual-energy x-ray absorptiometry (DXA), computed tomography (CT), bioelectrical impedance analysis (BIA), and magnetic resonance imaging (MRI) scan. However, they are not widely used due to their high cost and radioactivity and also because they are not feasible. Muscle strength is usually measured by handgrip strength and chair stand test, and physical performance is most frequently measured by gait speed, short physical performance battery and timed-up-and-go test. Thus, the complex and laborious operation process has limited the traditional screening of sarcopenia.

Therefore, alternative, simple, and economical biomarkers for diagnosing and monitoring sarcopenia are urgently needed. In 2012, Tetssuka et al. [7] proposed that the serum creatinine to serum cystatin C ratio (CCR) could be used as an indicator to evaluate the severity of amyotrophic lateral sclerosis (ALS). Kashani K. B et al. [8] validated the correlation between CCR and muscle mass, and they defined it as sarcopenia index (SI) in 2017. Its value has been verified successively in critically ill patients [9, 10], the older adults [11–13], organ transplant patients [14, 15], and patients with type 2 diabetes mellitus [16]. However, the value of SI in patients with EC has not yet been reported. There is no evidence that the value of SI is validated by big data. We assumed that SI could be used to screen for sarcopenia, and it is a prognostic factor among EC patients. Here, we aimed to test this hypothesis using two different models.

## Materials and methods

### Study design and participants

This study consisted of two phases, screening phase and validation phase. We explored the feasibility of SI in diagnosing sarcopenia using big data in the screening phase. Then, we validated the diagnostic value of SI and explored its prognostic value in clinical outcomes among patients with EC in the validation phase.

The national health and nutrition examination survey (NHANES) database (https://www.cdc.gov/nchs/nhanes) is a cross-sectional survey of nutrition assessment and health status based on the population of United States instead of cancer patients. It was initiated by the Centers for Disease Control and Prevention in 1971 using a multistage, probability-based, and stratified sampling design. About 10,000 participants have been interviewed and/or examined every 2 years, and the corresponding data have been released since 1999.

In this study, two survey cycles of participants in NHANES (1999–2000, 2001–2002) were used to investigate whether SI could be used to screen for sarcopenia in U.S. civilians. Linear correlations between SI and handgrip strength (HGS), appendicular skeletal muscle (ASM), and gait speed were evaluated. The participants were divided into three subgroups according to tertiles of the SI value. Differences in long-term survival among groups were compared. Restricted cubic spline (RCS) analysis was conducted to assess the potential non-linear or dose-response relationship between SI and risk for all-cause mortality.

Meanwhile, we identified patients who underwent esophagectomy at Jinling Hospital to validate the value of SI. Linear correlations between SI and skeletal muscle index (SMI), albumin, and prealbumin were assessed. The primary outcomes of interest were postoperative complications. Complications were defined according to the international consensus proposed by the Esophageal Complications Consensus Group (ECCG) [17] and classified according to the Clavien-Dindo classification (Supplementary Table 1) [18, 19]. Major complications were defined as Clavien-Dindo grade ≥ III. Overall complications were defined as the number of patients with one or more complications. The secondary outcome was long-term survival.

For participants whose data were extracted from NHANES, the inclusion criteria were as follows: (1) age ≥ 60 years; (2) with data for diagnosis of sarcopenia, including HGS, DXA, and gait speed. Participants were excluded when: (1) individuals had an amputation; (2) estimated glomerular filtration rate (eGFR) < 90 mL/min/1.73 m^2^; (3) individuals without SI data. For patients with EC in our institution, the adult patients (age ≥ 18) in whom serum creatinine and cystatin C were measured before esophagectomy from June 1, 2019 to June 30, 2021 were enrolled. Patients without abdominal CT scans data which were necessary to diagnose sarcopenia were excluded.

### Measurement of laboratory indicators

In the NHANES database, the analyses of biochemistry indicators were performed with a Hitachi Model 704 multichannel analyzer (Boehringer Mannheim Diagnostics, Indianapolis, IN). Serum creatinine was measured by the colorimetric method. Serum cystatin C was measured by the Dade Behring Nephelometer II (BNII) using an automated particle-enhanced nephelometric assay (PENIA) [20].

Serum creatinine and cystatin C levels of EC patients in our institution were measured using 7600 automatic biochemical analyzer (Hitachi, Tokyo, Japan) before esophagectomy. SI was defined as the ratio of serum creatinine (mg/mL) to cystatin C (mg/L): SI = (creatinine/cystatin C) × 100. Additionally, preoperative serum albumin and prealbumin levels of the included patients were measured.

### Diagnostic criteria for sarcopenia

For participants whose data were extracted from NHANES, sarcopenia was defined according to the latest version of criteria presented by the European Working Group on Sarcopenia in Older People (EWGSOP2) [4]. The optimal cut-off values of HGS and ASM were 27 kg and 20 kg for men, respectively, and 16 kg and 15 kg for women, respectively. The cut-off value of gait speed was 0.8 m/s. In the participants in NHANES during 1999–2002, knee extensor strength (KES) was measured using a Kin Com MP dynamometer (Chattanooga Group Inc., Chattanooga, Tennessee, USA). The maximum value of measurements was selected for analysis. According to the results of previous studies, KES was positively correlated with HGS (*r*=0.380, *p*<0.01) [21]. Thus, we calculated HGS using the following formula: HGS = (KES − 15.1)/0.65. ASM was measured by whole body DXA scan, which was performed with a Hologic QDR-4500A fan-beam densitometer (Hologic Inc., Bedford, Massachusetts, USA). The participants were asked to finish a 20-feet-long walk test at their usual pace, and gait speed was calculated using the following formula: Gait speed = 6.096/Time.

For patients with EC, the abdominal CT scan before surgery was used to calculate the skeletal muscle area (SMA) at the third lumbar vertebral level (L3) (Supplementary Figure 1). The software ImageJ® (version 1.53e) was utilized to delimit SMA [22]. SMI was defined as the ratio of SMA to squared height (SMI = SMA/height^2^). Low SMI was a component of sarcopenia and may be regarded as a suggestive indicator of sarcopenia. Patients with SMI < 40.8 cm^2^/m^2^ for men and SMI < 34.9 cm^2^/m^2^ for women were regarded as the presence of sarcopenia. We chose these criteria since these were the most frequently used criteria for Chinese population in previous studies [23].

### Follow-up of participants

All included individuals in the NHANES had a follow-up through December 31, 2015. Mortality status and the causes of death were extracted from the National Death Index records. Overall survival (OS) time was defined as the number of years from the examination at the mobile examination center (MEC) to the date of death or December 31, 2015. Likewise, for all included EC patients, follow-up was performed via phone interview before July 30, 2021. OS was defined as the number of months from hospital admission to the date of death or July 30, 2021.

### Statistical analyses

Continuous variables are presented as mean with standard deviation (SD) or median with interquartile range (IQR). Quantile–quantile plot and Shapiro–Wilk test were used to test the normality of data. Students’ *t* test or Wilcoxon rank-sum test was used to compare the difference among groups. Categorical variables are expressed by the quantity and proportion with χ2 tests or Fisher exact test. Scatter plots and Pearson's correlation coefficients were applied to evaluate linear correlations. Receiver operating characteristic (ROC) curves with area under curves (AUCs) were utilized to assess the ability of SI to screen for sarcopenia. The optimal cut-off values of SI for males and females were determined by calculating the Youden index (specificity + sensitivity − 1). A stepwise backward elimination method with a significance level of 0.05 was applied to establish the regression models. Variance inflation factor (VIF) was used to detect the severity of multicollinearity when constructing models. Models with VIF>5 referred to a significant multicollinearity [24]. Multivariable logistic regression models were used to identify independent predictive factors of postoperative complications. Kaplan–Meier curves were performed to estimate OS, and log-rank test was used to compare the difference between groups. Cox regression models were used to identify independent prognostic factors. RCS analysis was performed with four knots at the 5th, 35th, 65th, and 95th centiles. All analyses were performed by Stata 16.0 (Stata Corp LLC, USA). Sampling weight was considered when analyzing data in the NHANES database. A two-tailed *p*<0.05 was considered statistically significant.

## Results

### Characteristics of the study population

#### NHANES population

**Fig. 1:**
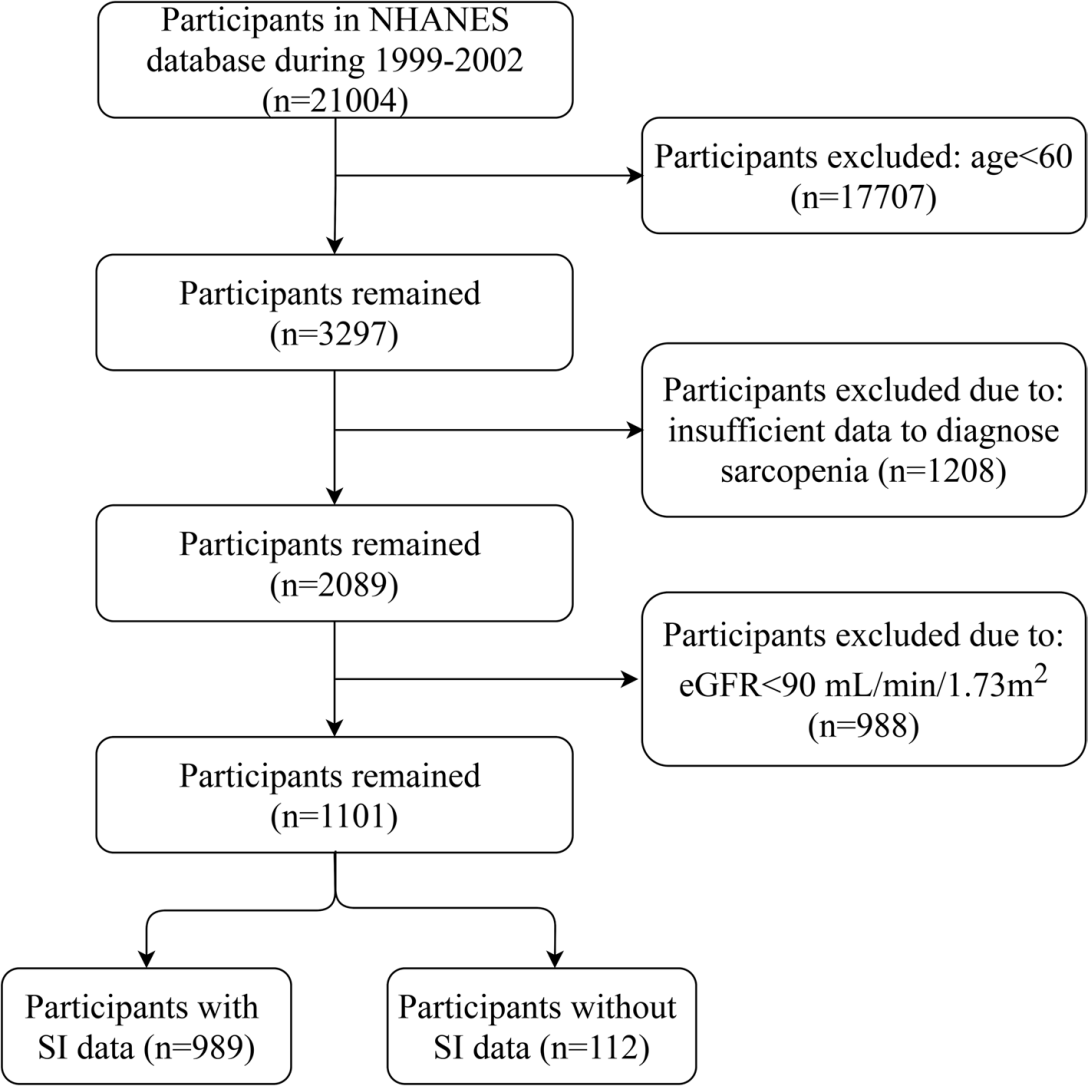
Flow chart of NHANES population selection

Data of 1,101 participants were extracted from the NHANES database, of which the SI of 112 individuals had not been evaluated (Fig. 1). To test the robustness of the results, we conducted sensitivity analysis, comparing the baseline characteristics between participants with and without SI data. A significant difference between two groups was not observed (Supplementary Table 2). The average age was 68.4 years, and 470 (47.52%) participants were male. According to the diagnostic criteria, 110 (11.1%) participants were diagnosed with sarcopenia, of which 41 (4.2%) participants were diagnosed with severe sarcopenia. The differences between the sarcopenia group and the non-sarcopenia group are presented in Table 1. Participants in the sarcopenia group were significantly older and had lower levels of BMI and SI than those in the non-sarcopenia group. Besides, body composition analyses indicated that not only skeletal muscle mass but also body fat of sarcopenia group were significantly lower. The differences in long-term survival among non-sarcopenia, sarcopenia, and severe sarcopenia groups were presented using Kaplan–Meier curve (Supplementary Figure 2). Compared with non-sarcopenia group, the median survival time of sarcopenia group and severe sarcopenia group were significantly shorter, and severe sarcopenia group had a poorest prognosis.

**Table 1:** Comparison of the baseline characteristics between non-sarcopenia and sarcopenia participants whose data were extracted from the NHANES

Variables	Non-sarcopenia (*n*=879)	Sarcopenia (*n*=110)	*p* value
Age, years	67.8±6.2	73.1±7.9	<0.001
Sex (male), n (%)	426 (48.5)	44 (40.0)	0.094
Race (white), n (%)	497 (56.5)	68 (61.8)	0.405
Body mass index, kg/m^2^	28.5±4.8	22.8±3.6	<0.001
Serum creatinine, mg/dL	0.69±0.14	0.64±0.14	<0.001
Serum cystatin C, mg/L	0.89±0.15	0.95±0.17	<0.001
SI	79.0±17.9	68.1±13.7	<0.001
Albumin, g/L	41.4±2.8	43.1±3.3	0.373
Total protein, g/L	74.6±4.6	75.2±5.1	0.225
Iron, umol/L	15.9±5.7	17.8±6.7	0.001
Uric acid, umol/L	312.8±80.6	278.9±66.4	<0.001
Cholesterol, mmol/L	265.3±49.4	263.0±58.7	0.769
Blood urea nitrogen, mmol/L	5.2±1.4	5.0±1.6	0.159
Bone mineral density, g/cm^2^	1.06±0.13	0.90±0.13	<0.001
Total fat, kg	28.9±9.0	20.4±6.7	<0.001
Total lean mass, kg	47.6±10.9	36.8±7.0	<0.001
Calf circumference, cm	37.4±3.5	32.6±3.1	<0.001
Arm circumference, cm	32.5±3.9	27.6±3.3	<0.001
Waist circumference, cm	100.1±12.9	87.7±11.4	<0.001
Thigh circumference, cm	50.7±5.6	43.4±4.7	<0.001
Triceps skinfold, mm	19.4±7.7	15.0±6.9	<0.001
ASM, kg	20.2±5.3	14.8±3.3	<0.001
ASMI, kg/m^2^	7.3±1.3	5.7±0.9	<0.001
Handgrip strength, kg	27.7±8.7	15.9±4.6	<0.001
Gait speed, m/s	0.99±0.23	0.94±0.33	0.042

Abbreviations: *SI* sarcopenia index, *ASM* appendicular skeletal muscle, *ASMI* appendicular skeletal muscle index (ASMI=ASM/height^2^)

#### Patients with EC

A total of 203 eligible patients with EC were identified from our institution, and their baseline characteristics are presented in Table 2. The prevalence of sarcopenia was 37.4%, including 62 males and 14 females. Compared with patients without sarcopenia, those who were diagnosed with sarcopenia were older (68.5 years vs. 65.9 years, *p*=0.038) and had a lower SI (59.2 vs. 68.1, *p*<0.001) and body mass index (BMI) (22.1 kg/m^2^ vs. 23.2 kg/m^2^, *p*=0.028).

**Table 2:** Main characteristics of the 178 patients with EC enrolled in Jinling Hospital

Variables	Mean/n	SD/%
Age, years	66.7	9.0
Sex		
Male	162	79.8
Female	41	20.2
Body mass index, kg/m^2^	22.6	3.3
T stage		
1	37	18.2
2	52	25.6
3	103	50.8
4	11	5.4
N stage		
0	102	50.2
1	68	33.5
2	29	14.3
3	4	2.0
Histology		
ESCC	180	88.7
EAC	14	6.9
Others	9	4.4
Serum creatinine, mg/dL	0.75	0.18
Serum cystatin C, mg/L	1.17	0.29
SI	64.7	12.0
Serum albumin, g/L	40.2	4.3
Serum prealbumin, mg/L	264.4	52.9
SMI, cm^2^/m^2^	40.9	6.3
Sarcopenia	76	37.4

Abbreviations: *SD* standard deviation, *ESCC* esophageal squamous carcinoma, *EAC* esophageal adenocarcinoma, *SI* sarcopenia index, *SMI* skeletal muscle index

### Exploration of the association between SI and sarcopenia using the NHANES database

**Fig. 2:**
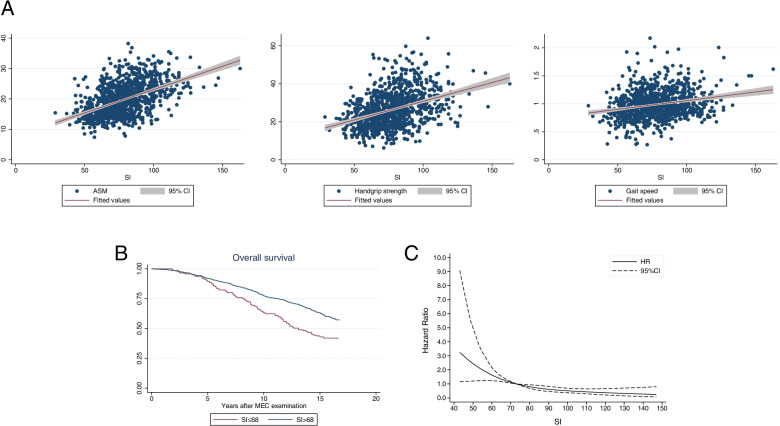
NHANES population: **A** Linear correlation between SI and ASM, HGS, and gait speed; **B** Kaplan-Meier curve stratified by SI≤68 and SI>68; **C** Non-linear relationship between SI and all-cause mortality risk using restricted cubic spline, y axis refers to hazard ratio, x axis refers to SI value

To investigate the association between SI and sarcopenia, we performed linear regressions. The Pearson’s correlation coefficients between SI and ASM, HGS, and gait speed were 0.487, 0.383, and 0.227, respectively (all *p*<0.001) (Fig. 2A). The participants were divided into three groups according to the tertiles of SI (tertile 1: SI ≤ 68, *n*=335; tertile 2: 69–84, *n*=331; tertile 3: SI ≥ 85, *n*=323). Multivariable logistic regression model weighting, which involved sampling weights of cystatin C (1999–2002) and adjusting them for gender and race, revealed that SI ≤ 68 was a predictive factor of sarcopenia (tertile 1 vs. tertile 3: OR=3.67, 95% CI: 1.52–8.87, *p*=0.004; tertile 2 vs. tertile 3: OR=1.79, 95% CI: 0.75–4.28, *p*=0.191; tertile 1 vs. tertile 2: OR=2.05, 95% CI: 1.15–3.64, *p*=0.014). Considering the linear relationship between age and SI (*r*= −0.24, *p*<0.001) and significant multicollinearity when including age (mean VIF=8.1), it was not included in the regression model.

The median follow-up time of all participants was 14.3 years (range: 0–16.7 years). The Kaplan–Meier curve indicated that the participants with SI ≤ 68 had a poorer long-term survival than those with SI > 68 (Fig. 2B). The multivariable Cox proportional regression model adjusted for race, gender, and BMI revealed that SI was an independent prognostic factor of OS (adjusted HR=2.14, 95% CI: 1.71–2.68, *p*<0.001). Meanwhile, the RCS plot (reference value: 68) showed that the all-cause mortality risk gradually decreased with the increase in SI (Fig. 2C). Therefore, SI has the potentiality to screen for sarcopenia.

### Validation of the value of SI among EC patients

#### Primary outcomes

**Fig. 3:**
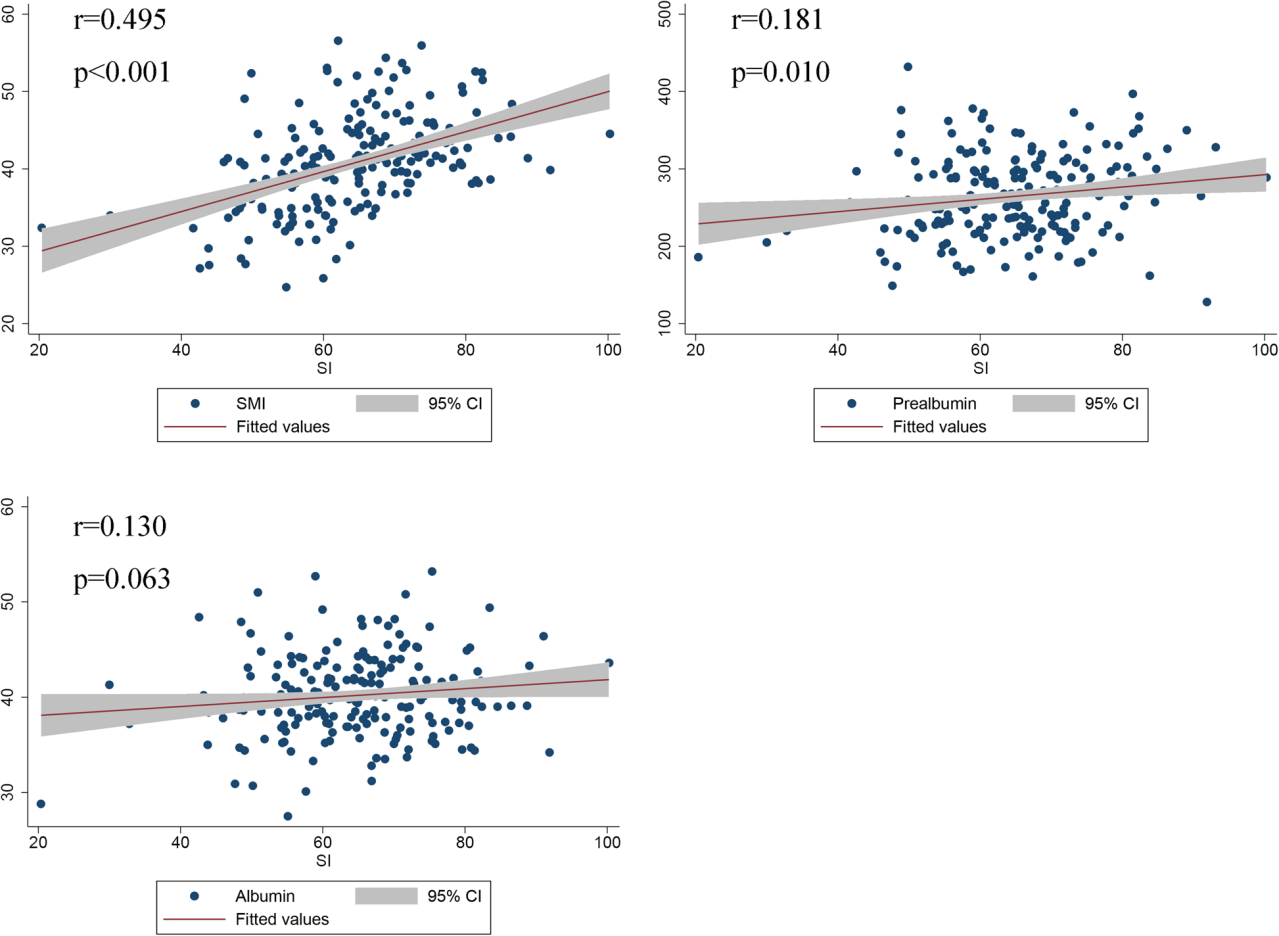
Linear correlation between SI and SMI, albumin, and prealbumin among EC patients

**Fig. 4:**
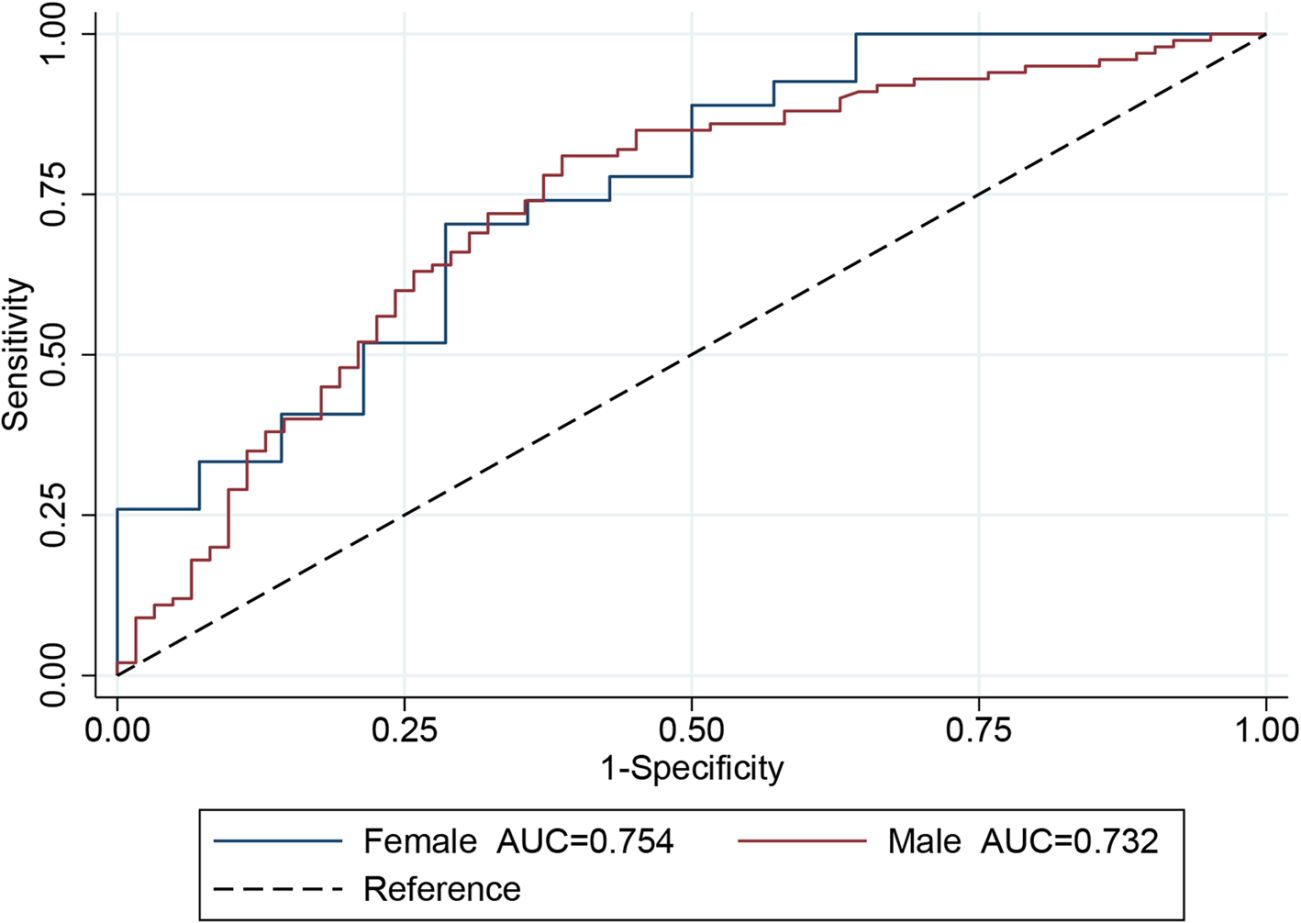
ROC curves of SI in screening sarcopenia among EC patients

SI showed a significant linear correlation between SMI (*r*=0.495, *p*<0.001) and prealbumin (*r*=0.181, *p*=0.010) but not with albumin (*r*=0.130, *p*=0.063), indicating that SI was an ideal alternative biomarker to screen for sarcopenia (Fig. 3). The ROC curves indicated that SI had a good diagnostic efficacy for sarcopenia whether in males (AUC=0.732) or females (AUC=0.754) (Fig. 4). By calculating the Youden index, the optimal cut-off values of SI for males and females were 62 and 55, respectively. Thus, male patients with SI < 62 and female patients with SI < 55 were defined as the high sarcopenia risk group, and the other patients were considered as the low sarcopenia risk group. The comparison of characteristics between the two groups is presented in Table 3. Patients in the high sarcopenia risk group had a significantly lower level of BMI and SMI, as well as higher incidences of overall complications, major complications, pneumonia, and anastomotic leakage. In addition, patients in high sarcopenia risk group had longer length of hospital stay. Significant differences in the incidence of anastomotic stenosis, and incision infection and myocardia arrhythmia were not observed between the two groups.

**Table 3:** Comparison of characteristics among patients stratified by the risk of sarcopenia

Variables	Low sarcopenia risk group (*n*=128)	High sarcopenia risk group (*n*=75)	*p* value
Age, years	65.8±9.5	68.7±7.8	0.023
Sex (male), n (%)	105 (82.0)	57 (76.0)	0.302
Body mass index, kg/m^2^	23.1±3.0	21.9±3.7	0.015
Serum albumin, g/L	40.5±4.1	39.6±4.6	0.125
Serum prealbumin, mg/L	265.3±49.4	263.0±58.7	0.769
SMI, cm^2^/m^2^	43.0±5.3	37.2±6.1	<0.001
Sarcopenia, n (%)	28 (21.9)	48 (64.0)	<0.001
SI	71.6±8.2	53.0±7.4	<0.001
Complications			
Overall complications, n (%)	39 (30.5)	43 (57.3)	<0.001
Major complications, n (%)	12 (9.4)	15 (20.0)	0.031
Pneumonia, n (%)	17 (13.3)	24 (32.0)	0.001
Anastomotic leakage, n (%)	11 (8.6)	15 (20.0)	0.019
Anastomotic stenosis, n (%)	6 (4.7)	4 (5.3)	0.837
Incision infection, n (%)	11 (8.6)	7 (9.3)	0.858
Myocardia arrhythmia, n (%)	9 (7.0)	7 (9.3)	0.557
LOS of hospital, median (IQR), day	9.5 (8–15)	13 (10–18)	<0.001

Abbreviations: *SMI* skeletal muscle index, *SI* sarcopenia index, *LOS* length of stay, *IQR* interquartile ranges

Multivariable logistic regression models adjusted for gender, BMI, T stage, N stage, and histology showed that patients with low SI was independently associated with overall complications (adjusted OR=3.50, 95% CI: 1.85–6.61, *p*<0.001), major complications (adjusted OR=2.73, 95% CI: 1.07–6.99, *p*=0.036), pneumonia (adjusted OR=3.67, 95% CI: 1.59–8.47, *p*=0.002), and anastomotic leakage (adjusted OR=2.99, 95% CI: 1.10–8.12, *p*=0.031).

#### Long-term outcomes

**Fig. 5:**
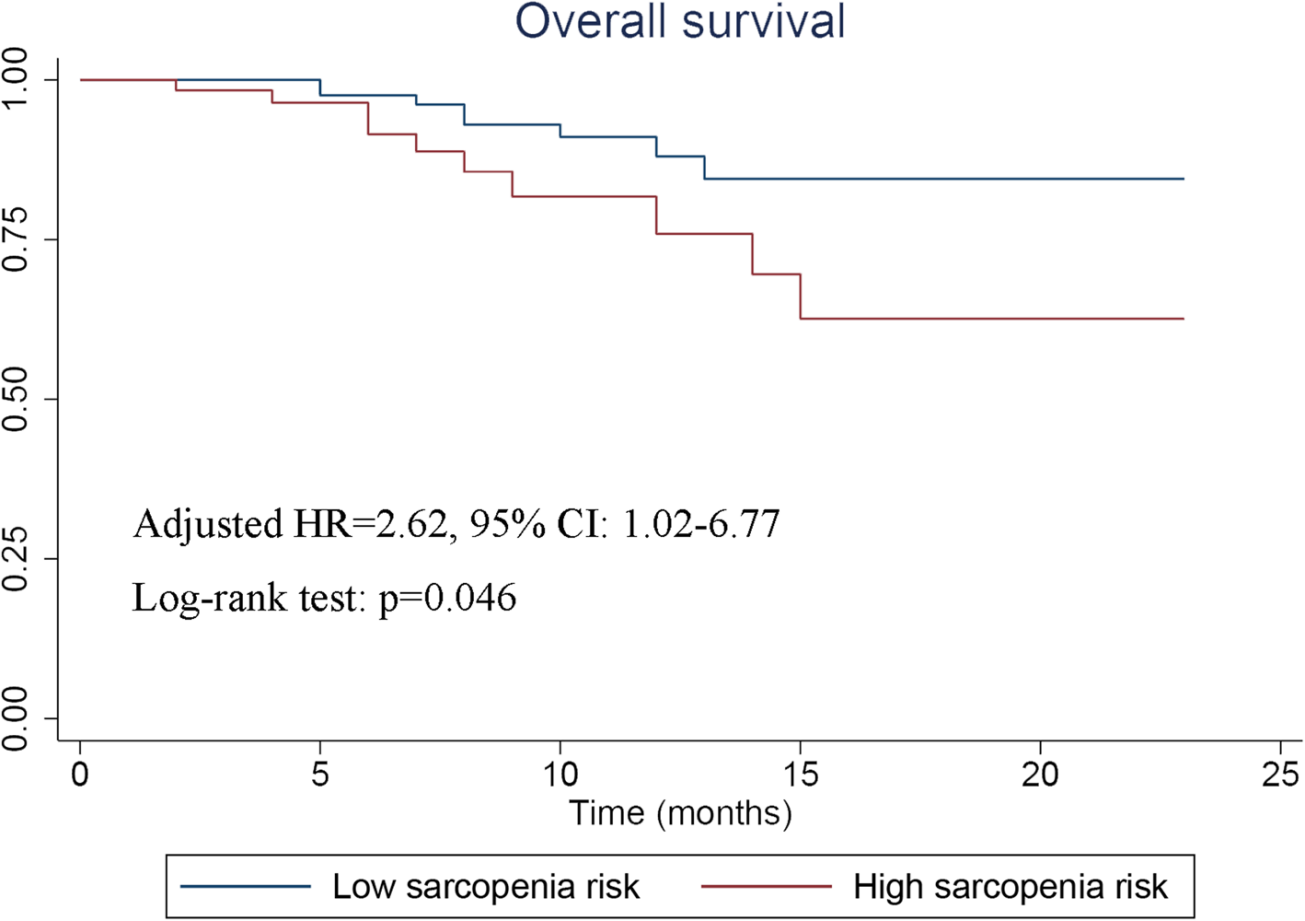
Kaplan-Meier curve stratified by risk of sarcopenia among EC patients

The median follow-up time of patients with EC was 7 months (range: 0–23 months). Kaplan–Meier curve was plotted to compare survival differences (Fig. 5). The result of Cox regression model revealed that SI was an independent prognostic factor of OS in patients with EC (adjusted HR=2.62, 95% CI: 1.02–6.77, *p *= 0.046).

#### Comparison of SI and other nutritional indexes

Further analyses were conducted to compare the value of SI and other indicators. The diagnostic values of nutritional indexes to binary outcome were evaluated by ROC curves with AUC values. The prognostic value of nutritional indexes to overall survival was assessed by time-dependent ROC curve with AUC value (Supplementary Table 3). The results indicated that SI had a considerable performance with SMI and better than BMI, albumin and prealbumin.

## Discussion

Traditionally, sarcopenia is evaluated by skeletal muscle mass, muscle strength, and physical performance. We verified that SI could be used to screen for sarcopenia from two aspects. SI was not only significantly associated with ASM, HGS, and gait speed but also with SMI. Thus, SI could be a surrogate biomarker for identifying sarcopenia in EC patients. Serum creatinine and cystatin C were widely applied for evaluating renal function. Serum creatinine is mostly produced from creatine phosphate during the metabolism of skeletal muscle; thus, patients with decreased muscle mass have a lower level of creatinine [25]. Meanwhile, serum cystatin C is a small-sized, nonionic protein produced by nucleated cells at a constant rate and not influenced by muscle metabolism [26]. Based on these features, SI was calculated to better evaluate the muscle mass, and it showed the potential for screening sarcopenia. We also found that patients with sarcopenia had a higher level of serum iron and lower level of uric acid. As an endogenous antioxidant, higher serum uric acid levels are associated with better muscle function, and iron accumulation was associated with impaired muscle regeneration in the process of oxidative stress [27, 28].

The optimal cut-off value of SI to screen for sarcopenia in patients with EC was determined. Patients in the high sarcopenia risk group had a higher incidence of postoperative complications, especially inflammation-mediated complications, such as pneumonia and anastomotic leakage. Former researchers proposed that inflammation was associated with increased serum cystatin C and decreased creatinine [29], which might be one of the potential reasons why SI could predict inflammation-related complications. Moreover, SI was found to be a prognostic factor of long-term survival. Patients who undergo esophagectomy always suffer from weight loss and malnutrition due to alteration of the dietary pattern. Previous studies have reported that enteral nutrition support could not only improve patients’ nutritional status and immune function but also preserve the skeletal muscle mass [30, 31]. Family enteral nutrition was reported to reduce the risk of malnutrition and incidence of complications after esophagectomy [32]. Hence, early screening of sarcopenia is essential.

Since the introduction of SI, its application prospect has been evaluated in various diseases, as it is cheap, effective, objective and replicable. A retrospective study by Jung et al. indicated that SI could predict the 30- and 90-day mortality of patients who underwent continuous kidney replacement therapy [33]. The results were inconsistent among older individuals. Tan et al. [12] recommended SI as a screening tool for low HGS in community-dwelling older individuals, while He et al. [11] did not recommend it. In 2020, Ulmann G. et al. [34] validated the efficacy of SI in 44 cancer patients and found it to be better than that of BIA. Gao et al. [35] found that SI could predict postoperative complications after gastric cancer surgery. To our knowledge, the value of SI among EC patients was assessed for the first time. Using the NHANES database and EC patients, our study enriched the application field of SI. Recently, several other indicators based on serum creatinine and cystatin C were reported. In the studies by Yang et al. [36] and Fu et al. [37], the new version of SI, serum creatinine × cystatin C-based eGFR, also showed its potential to screen for low muscle mass or sarcopenia in cancer patients. Following this, Nishida et al. [38] proposed that the creatinine/ (cystatin C × weight) ratio was associated with SMI in patients with T2DM, considering insulin sensitivity.

This study has several limitations. HGS was estimated according to KES, which might have caused bias, although HGS was significantly associated with KES. Besides, serum cystatin C was hardly ever measured in EC patients in our institution before 2019; thus, the follow-up time was not long enough. Use of the SMI derived from CT scan at the L3 level to diagnose sarcopenia was internationally accepted by researchers, but it took only muscle quantity rather than muscle quality into account. In our future studies, we will also prospectively collect data of muscle strength and function to verify our findings. In addition, the major histological subtype of EC patients was squamous cell carcinoma which is known to have an increased rate of sarcopenia [39]. Subgroup analyses should be conducted to test the value of SI in different histological subtypes.

## Conclusion

SI could be a simple, economic, and effective screening tool for sarcopenia in patients with EC, and it is a helpful prognostic factor of postoperative complications and long-term survival.

## Supplementary Information

**Additional file 1: Supplementary Table 1.** Detailed information on Clavien-Dindo classification. **Supplementary Table 2.** Comparison of characteristics between participants with or without SI in the NHANES database. **Supplementary Table 3.** Comparison of predictive performance of nutritional indicators on outcomes using AUC values. **Supplementary Figure 1.** Delimitation of skeletal muscle area (SMA) at L3 level among EC patients. **Supplementary Figure 2.** Kaplan-Meier curve of NHANES population stratified by sarcopenia, severe sarcopenia, and non-sarcopenia.

## Data Availability

The datasets generated and/or analysed during the current study are available in the NHANES database, (https://wwwn.cdc.gov/nchs/nhanes/continuousnhanes/default.aspx?BeginYear=1999, https://wwwn.cdc.gov/nchs/nhanes/continuousnhanes/default.aspx?BeginYear=2001). The data of EC patients in our hospital are not publicly available due to privacy and data sharing issues but are available from the corresponding author on reasonable request.

## References

[1] Sung H, Ferlay J, Siegel R, Laversanne M, Soerjomataram I, Jemal A, Bray F (2021). Global Cancer Statistics 2020: GLOBOCAN Estimates of Incidence and Mortality Worldwide for 36 Cancers in 185 Countries. CA: A Cancer J Clin.

[2] Wang P, Xu L, Chen X, Xu L, Yu Y, Zhang R, Sun H, Wu H, Li Y (2020). Sarcopenia and Short-Term Outcomes After Esophagectomy: A Meta-analysis. Ann Surg Oncol.

[3] Nakashima Y, Saeki H, Nakanishi R, Sugiyama M, Kurashige J, Oki E, Maehara Y (2018). Assessment of Sarcopenia as a Predictor of Poor Outcomes After Esophagectomy in Elderly Patients With Esophageal Cancer. Ann Surg.

[4] Cruz-Jentoft A, Bahat G, Bauer J, Boirie Y, Bruyère O, Cederholm T, Cooper C, Landi F, Rolland Y, Sayer A (2019). Sarcopenia: revised European consensus on definition and diagnosis. Age Ageing.

[5] Chen L, Woo J, Assantachai P, Auyeung T, Chou M, Iijima K, Jang H, Kang L, Kim M, Kim S (2020). Asian Working Group for Sarcopenia: 2019 Consensus Update on Sarcopenia Diagnosis and Treatment. J Am Med Dir Assoc.

[6] Fielding R, Vellas B, Evans W, Bhasin S, Morley J, Newman A, Abellan van Kan G, Andrieu S, Bauer J, Breuille D (2011). Sarcopenia: an undiagnosed condition in older adults. Current consensus definition: prevalence, etiology, and consequences. International working group on sarcopenia. J Am Med Dir Assoc.

[7] Tetsuka S, Morita M, Ikeguchi K, Nakano I. Creatinine/cystatin C ratio as a surrogate marker of residual muscle mass in amyotrophic lateral sclerosis. Neurology and Clinical. Neuroscience. 2013;1(1):32-37.

[8] Kashani K, Frazee E, Kukrálová L, Sarvottam K, Herasevich V, Young P, Kashyap R, Lieske J (2017). Evaluating Muscle Mass by Using Markers of Kidney Function: Development of the Sarcopenia Index. Crit Care Med.

[9] Barreto E, Poyant J, Coville H, Dierkhising R, Kennedy C, Gajic O, Nystrom E, Takahashi N, Moynagh M, Kashani K (2019). Validation of the sarcopenia index to assess muscle mass in the critically ill: A novel application of kidney function markers. Clin Nutri (Edinburgh, Scotland).

[10] Barreto E, Kanderi T, DiCecco S, Lopez-Ruiz A, Poyant J, Mara K, Heimgartner J, Gajic O, Rule A, Nystrom E (2019). Sarcopenia Index Is a Simple Objective Screening Tool for Malnutrition in the Critically Ill. JPEN.

[11] He Q, Jiang J, Xie L, Zhang L, Yang M (2018). A sarcopenia index based on serum creatinine and cystatin C cannot accurately detect either low muscle mass or sarcopenia in urban community-dwelling older people. Sci Rep.

[12] Tan L, Li R, Hu X, Zhu Y, Bao T, Zuo Y, Yang M (2020). Serum creatinine/cystatin C ratio as a case-finding tool for low handgrip strength in Chinese middle-aged and older adults. Sci Rep.

[13] Tang T, Zhuo Y, Xie L, Wang H, Yang M (2020). Sarcopenia index based on serum creatinine and cystatin C is associated with 3-year mortality in hospitalized older patients. Sci Rep.

[14] Kashani K, Sarvottam K, Pereira N, Barreto E, Kennedy C (2018). The sarcopenia index: A novel measure of muscle mass in lung transplant candidates. Clin Transplant.

[15] Yanishi M, Kinoshita H, Tsukaguchi H, Kimura Y, Koito Y, Sugi M, Matsuda T (2019). The creatinine/cystatin C ratio provides effective evaluation of muscle mass in kidney transplant recipients. Int Urol Nephrol.

[16] Osaka T, Hamaguchi M, Hashimoto Y, Ushigome E, Tanaka M, Yamazaki M, Fukui M (2018). Decreased the creatinine to cystatin C ratio is a surrogate marker of sarcopenia in patients with type 2 diabetes. Diab Res Clin Pract.

[17] Low D, Alderson D, Cecconello I, Chang A, Darling G, D’Journo X, Griffin S, Hölscher A, Hofstetter W, Jobe B (2015). International Consensus on Standardization of Data Collection for Complications Associated With Esophagectomy: Esophagectomy Complications Consensus Group (ECCG). Ann Surg.

[18] Dindo D, Demartines N, Clavien P (2004). Classification of surgical complications: a new proposal with evaluation in a cohort of 6336 patients and results of a survey. Ann Surg.

[19] Clavien P, Barkun J, de Oliveira M, Vauthey J, Dindo D, Schulick R, de Santibañes E, Pekolj J, Slankamenac K, Bassi C (2009). The Clavien-Dindo classification of surgical complications: five-year experience. Ann Surg.

[20] Finney H, Newman D, Gruber W, Merle P, Price C (1997). Initial evaluation of cystatin C measurement by particle-enhanced immunonephelometry on the Behring nephelometer systems (BNA, BN II). Clin Chem.

[21] Yasuda T (2019). Field-Based Simplified Approach of Evaluating Knee Extensor Muscle Strength and Size in University Freshmen Women. J Sport Rehabil.

[22] Teigen L, Kuchnia A, Nagel E, Deuth C, Vock D, Mulasi U, Earthman C (2018). Impact of Software Selection and ImageJ Tutorial Corrigendum on Skeletal Muscle Measures at the Third Lumbar Vertebra on Computed Tomography Scans in Clinical Populations. JPEN.

[23] Su H, Ruan J, Chen T, Lin E, Shi L (2019). CT-assessed sarcopenia is a predictive factor for both long-term and short-term outcomes in gastrointestinal oncology patients: a systematic review and meta-analysis. Cancer Imag.

[24] Marcoulides K, Raykov T (2019). Evaluation of Variance Inflation Factors in Regression Models Using Latent Variable Modeling Methods. Educ Psychol Meas.

[25] Haines R, Zolfaghari P, Wan Y, Pearse R, Puthucheary Z, Prowle J (2019). Elevated urea-to-creatinine ratio provides a biochemical signature of muscle catabolism and persistent critical illness after major trauma. Intens Care Med.

[26] Randers E, Erlandsen E (1999). Serum cystatin C as an endogenous marker of the renal function--a review. CCLM.

[27] Molino-Lova R, Sofi F, Pasquini G, Vannetti F, Del Ry S, Vassalle C, Clerici M, Sorbi S, Macchi C (2017). Higher uric acid serum levels are associated with better muscle function in the oldest old: Results from the Mugello Study. Eur J Intern Med.

[28] Alves F, Kysenius K, Caldow M, Hardee J, Crouch P, Ayton S, Bush A, Lynch G, Koopman R (2021). Iron accumulation in skeletal muscles of old mice is associated with impaired regeneration after ischaemia-reperfusion damage. J Cachexia Sarcopenia Muscle.

[29] Svensson A, Kvitting J, Kovesdy C, Cederholm I, Szabó Z (2016). Changes in serum cystatin C, creatinine, and C-reactive protein after cardiopulmonary bypass in patients with normal preoperative kidney function. Nephrology (Carlton, Vic).

[30] Ding H, Xu J, You J, Qin H, Ma H (2020). Effects of enteral nutrition support combined with enhanced recovery after surgery on the nutritional status, immune function, and prognosis of patients with esophageal cancer after Ivor-Lewis operation. J Thorac Dis.

[31] Kita R, Miyata H, Sugimura K, Tanaka K, Makino T, Yamashita K, Yamasaki M, Motoori M, Shiraishi O, Kimura Y (2021). Clinical effect of enteral nutrition support during neoadjuvant chemotherapy on the preservation of skeletal muscle mass in patients with esophageal cancer. Clin Nutri (Edinburgh, Scotland).

[32] Chen T, Jiang W, He G (2021). Effect of family enteral nutrition on nutritional status in elderly patients with esophageal carcinoma after minimally invasive radical surgery: a randomized trial. Ann Palliat Med.

[33] Jung C, Joo Y, Kim H, Han S, Yoo T, Kang S, Park J (2021). Creatinine-Cystatin C Ratio and Mortality in Patients Receiving Intensive Care and Continuous Kidney Replacement Therapy: A Retrospective Cohort Study. Am J Kidney Dis.

[34] Ulmann G, Kaï J, Durand J, Neveux N, Jouinot A, De Bandt J, Goldwasser F, Cynober L (2021). Creatinine-to-cystatin C ratio and bioelectrical impedance analysis for the assessement of low lean body mass in cancer patients: Comparison to L3-computed tomography scan. Nutrition (Burbank, Los Angeles County, Calif).

[35] Gao J, Liang H, Qian Y, Pan J, Liu W, Qi W, Zhou W, Ge X, Wang X (2021). Creatinine-to-cystatin C ratio as a marker of skeletal muscle mass for predicting postoperative complications in patients undergoing gastric cancer surgery. Ann Palliat Med.

[36] Yang J, Zhang T, Feng D, Dai X, Lv T, Wang X, Gong J, Zhu W, Li J (2019). A new diagnostic index for sarcopenia and its association with short-term postoperative complications in patients undergoing surgery for colorectal cancer. Colorectal Dis.

[37] Fu X, Tian Z, Wen S, Sun H, Thapa S, Xiong H, Liu H, Li L, Yu S (2021). A new index based on serum creatinine and cystatin C is useful for assessing sarcopenia in patients with advanced cancer. Nutrition (Burbank, Los Angeles County, Calif).

[38] Nishida K, Hashimoto Y, Kaji A, Okamura T, Sakai R, Kitagawa N, Osaka T, Hamaguchi M, Fukui M (2020). Creatinine/(cystatin C × body weight) ratio is associated with skeletal muscle mass index. Endocr J.

[39] Elliott J, Doyle S, Murphy C, King S, Guinan E, Beddy P, Ravi N, Reynolds J (2017). Sarcopenia: Prevalence, and Impact on Operative and Oncologic Outcomes in the Multimodal Management of Locally Advanced Esophageal Cancer. Ann Surg.

