# Supplementary material for: Serum creatinine/cystatin C ratio as a screening tool for sarcopenia and prognostic indicator for patients with esophageal cancer
Source: BMC Geriatr. 2022 Mar 15;22:207. doi: 10.1186/s12877-022-02925-8 (PMC8922862; doi:10.1186/s12877-022-02925-8)
Supplement: Supplementary file 1 — Additional file 1: Supplementary Table 1. Detailed information on Clavien-Dindo classification. Supplementary Table 2. Comparison of characteristics between participants with or without SI in the NHANES database. Supplementary Table 3. Comparison of predictive performance of nutritional indicators on outcomes using AUC values. Supplementary Figure 1. Delimitation of skeletal muscle area (SMA) at L3 level among EC patients. Supplementary Figure 2. Kaplan-Meier curve of NHANES population stratified by sarcopenia, severe sarcopenia, and non-sarcopenia. [file 12877_2022_2925_MOESM1_ESM.pdf]

Supplementary Table 1. Detailed information on Clavien-Dindo classification

**Grades**	**Definition**
**I**	1. Any deviation from the normal postoperative course without the need for pharmacological treatment or surgical, endoscopic and radiological interventions.<br>2. Acceptable therapeutic regimens are: drugs as antiemetics, antipyretics, analgesics, diuretics and electrolytes and physiotherapy.<br>3. This grade also includes wound infections opened at the bedside.
**II**	1. Requiring pharmacological treatment with drugs other than such allowed for grade I complications.<br>2. Blood transfusions and total parenteral nutrition are also included.
**III**	Blood transfusions and total parenteral nutrition are also included.
**IIIa**	Intervention not under general anesthesia
**IIIb**	Intervention under general anesthesia
**IV**	Life-threatening complications (including central nervous system complications) * requiring IC/ICU-management
**IVa**	Single organ dysfunction (including dialysis)
**IVb**	Multiple organ dysfunction
**V**	Death

Notes: * means brain hemorrhage, ischemic stroke, subarachnoid bleeding, but excluding transient ischemic attacks; IC: intermediate care; ICU: intensive care unit

Supplementary Table 2. Comparison of characteristics between participants with or without SI in the NHANES database

Variables	Participants with SI (n=989)	Participants without SI (n=112)	p value
Age, year	68.4±6.6	69.3±6.5	0.185
Sex (male), n (%)	470 (47.5)	61 (54.5)	0.164
Race (White), n (%)	514 (52.0)	51 (45.5)	0.197
Body mass index, kg/m^2	27.9±5.1	27.6±5.9	0.522
Bone mineral density, g/cm^2	1.05±0.13	1.06±0.15	0.408
Total fat, kg	28.0±9.1	27.6±10.9	0.620
Total lean mass, kg	46.4±11.1	46.4±10.4	0.993
Calf circumference, cm	36.9±3.7	36.6±4.3	0.493
Arm circumference, cm	32.0±4.1	31.9±4.9	0.868
Waist circumference, cm	98.8±13.2	98.0±13.2	0.541
Thigh circumference, cm	49.9±5.9	50.5±7.2	0.312
Triceps skinfold, mm	18.9±7.8	18.1±8.6	0.351
ASM, kg	19.6±5.4	20.0±5.3	0.481
ASMI, kg/m^2	7.1±1.4	7.2±1.4	0.570
Handgrip strength, kg	26.4±9.2	25.3±9.5	0.213
Gait speed, m/s	0.98±0.25	1.01±0.24	0.336
Sarcopenia, n (%)	110 (11.1)	14 (12.5)	0.662

Abbreviations: SI, sarcopenia index; NHANES, national health and nutritional examination survey; ASM, appendicular skeletal muscle; ASMI, appendicular skeletal muscle index ($ASMI=ASM/height^2$).

Supplementary Table 3. Comparison of predictive performance of nutritional indicators on outcomes using AUC values

	SI	SMI	BMI	Albumin	Prealbumin
Major complications	0.776	0.720	0.509	0.495	0.620
AL	0.803	0.762	0.642	0.584	0.718
Pneumonia	0.821	0.758	0.559	0.561	0.650
Overall survival	0.713	0.680	0.602	0.541	0.547

Abbreviations: AUC, area under curve; SI, sarcopenia index; SMI, skeletal muscle index; BMI, body mass index; AL, anastomosis leakage.

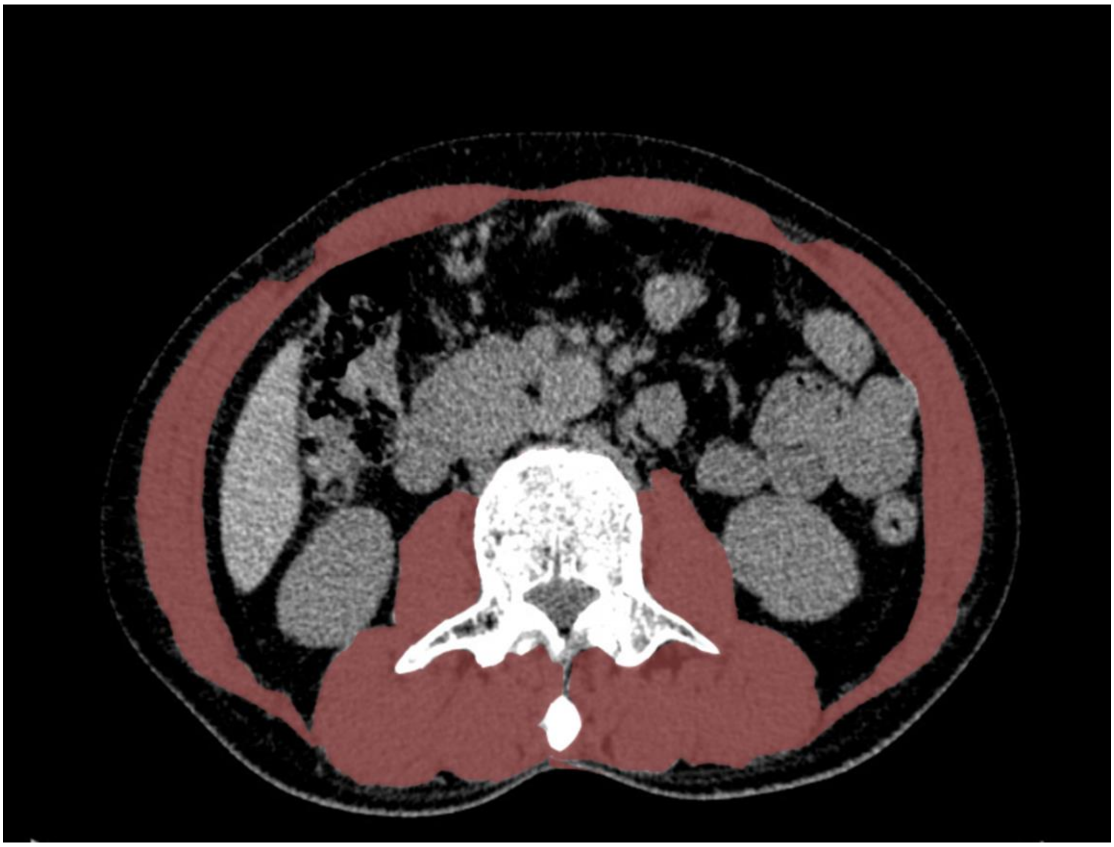

Supplementary Figure 1. Delimitation of skeletal muscle area (SMA) at L3 level among EC patients.

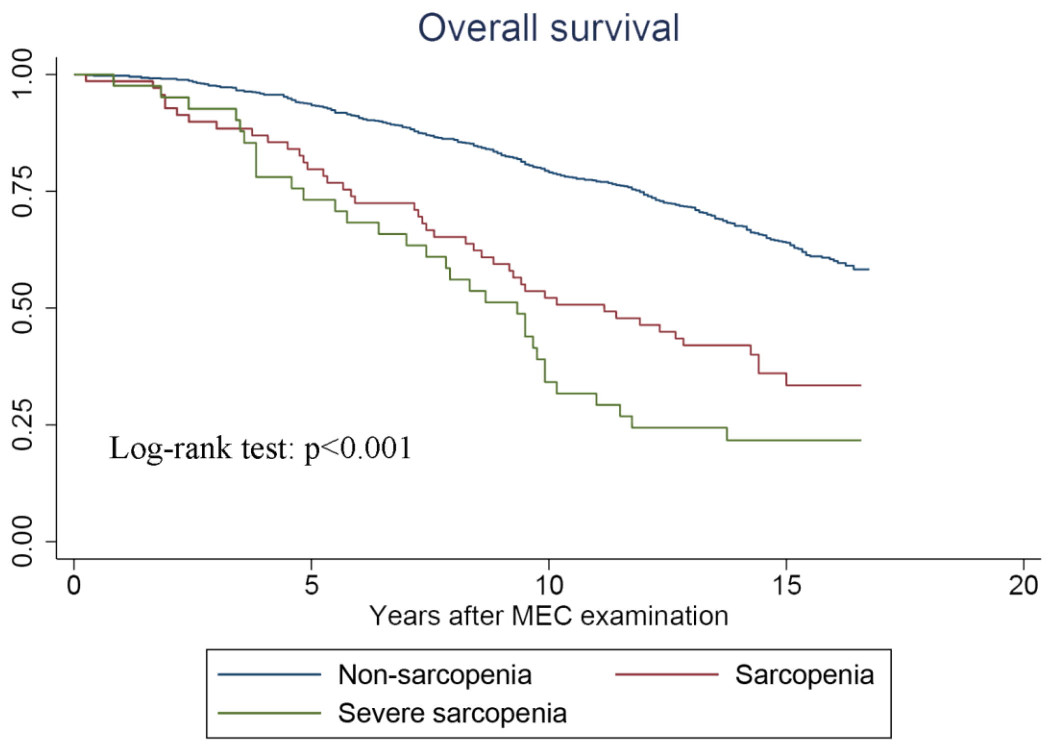

Supplementary Figure 2. Kaplan-Meier curve of NHANES population stratified by sarcopenia, severe sarcopenia, and non-sarcopenia.
